# Mature cystic teratoma mimicking a tailgut cyst in an adolescent female: a case report

**DOI:** 10.1093/jscr/rjae719

**Published:** 2024-11-25

**Authors:** Safaa Abatli, Yazan AlHabil, Mohammed Shawkat Hamad, Yousef Abulibdeh

**Affiliations:** Surgery Department, Darwish Nazal Governmental Hospital, Ministry of Health, Darwish Nazal Hospital St., Qalqileyah 00970, Palestine; Department of Biomedical Sciences, Faculty of Medicine and Health Sciences, An-Najah National University, P.O. Box. 7, Nablus 00970, Palestine; Faculty of Medicine and Health Sciences, Department of Medicine, An-Najah National University, Old Campus Street 7, Nablus 00970, Palestine; Surgery Department, Darwish Nazal Governmental Hospital, Ministry of Health, Darwish Nazal Hospital St., Qalqileyah 00970, Palestine; Faculty of Medicine and Health Sciences, Department of Medicine, An-Najah National University, Old Campus Street 7, Nablus 00970, Palestine; Surgery Department, Darwish Nazal Governmental Hospital, Ministry of Health, Darwish Nazal Hospital St., Qalqileyah 00970, Palestine; Faculty of Medicine and Health Sciences, Department of Medicine, An-Najah National University, Old Campus Street 7, Nablus 00970, Palestine

**Keywords:** tailgut cyst, adolescent female, presacral tumor

## Abstract

Presacral tumors are uncommon, particularly in the pediatric population, and can arise from various germ cell types during embryologic development. Tailgut cysts, or retrorectal cystic hamartomas, represent rare congenital anomalies resulting from defective regression of hindgut remnants. We present a unique case of a 13-year-old female with pelvic symptoms, initially suspected to have a tailgut cyst based on imaging findings. However, surgical exploration revealed a mature cystic teratoma, a rare occurrence in this age group. Surgical excision was performed using an anterior approach, revealing adhesions and necessitating meticulous dissection for complete removal. Histopathological examination of the mass unexpectedly confirmed a mature cystic teratoma, characterized by a fibrovascular cyst wall containing smooth muscle and lobules resembling salivary acini, the cyst’s surface exhibited squamous and respiratory-type epithelium. The accurate diagnosis of presacral masses, rather than relying solely on diagnostic measures, underscores the importance of prioritizing surgical exploration for definitive assessment and management.

## Introduction

The presacral space, located between the rectum and the lumbosacral spine, develops from various embryonic germ cell types and can harbor a range of tumors. Presacral tumors are uncommon, occurring at rates of 1.4 to 6.3 cases per year, primarily affecting individuals aged 30 to 50 years. Pediatric presacral masses are even rarer [1].

Tailgut cysts, also known as retrorectal cystic hamartomas, arise from remnants of the hindgut that fail to regress properly during embryonic development, with an overall incidence of ~1 in 40 000 [2]. These cysts are typically found in the retrorectal or presacral space, characterized anatomically by specific boundaries. Histologically, they exhibit epithelial features derived from embryonic cells, presenting as multicystic formations containing diverse epithelial types [3]. Clinical manifestations can vary from asymptomatic to nonspecific symptoms, often leading to delayed diagnosis, particularly in females aged 40–60 years [2].

Mature cystic teratomas (MCTs), or ovarian dermoid cysts, are common benign tumors in young women, frequently asymptomatic or causing pelvic pain. They originate from germ cells and consist of tissues from ectoderm, mesoderm, and endoderm, displaying diverse histological compositions. While most cases are benign, a small percentage may undergo malignant transformation, especially in postmenopausal women [4].

Herein, we report a rare case of a 13-year-old adolescent female presenting with a mature cystic teratoma initially mistaken for a tailgut cyst.

## Case presentation

A 13-year-old female patient presented to the hospital with complaints of pelvic heaviness, a sensation of incomplete defecation, and multiple episodes of urine retention. She mentioned that the symptoms worsened with prolonged sitting and standing. She denied any history of rectal bleeding, abdominal pain, or weight loss. She has unremarkable past medical and surgical histories.

**Figure 1:**
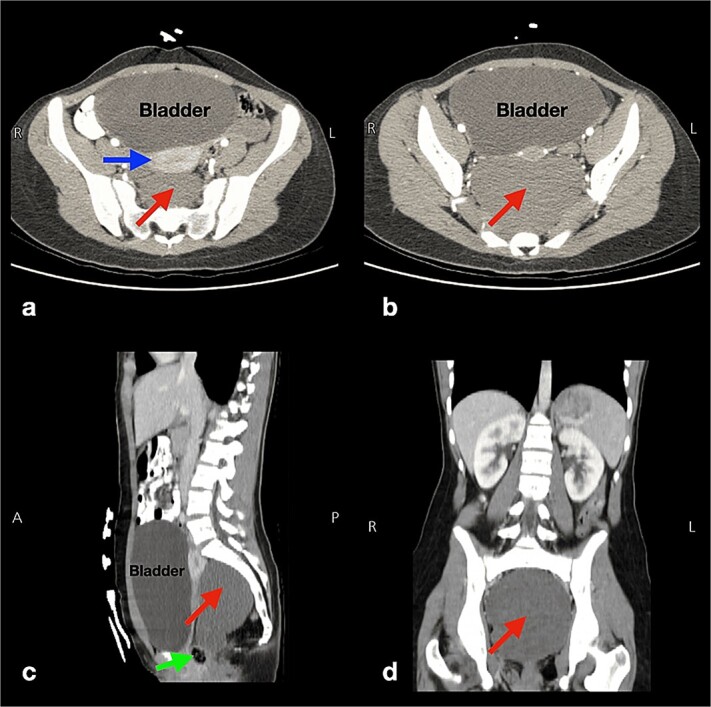
Multi-plane (a,b: axial and c: sagittal, d: coronal) abdominopelvic computed tomography scans displaying a well-defined cystic lesion, with fluid attenuation, and of biloculated nature measuring 11 × 10.5 × 8 cm (arrows in pictures a, b, c, d). This lesion has no soft tissue components or calcifications and is located in the presacral region near the sigmoid colon, rectum (arrow in picture c), and the uterus anteriorly (horizontal arrow in picture a), and both iliac vessels laterally. Also, the bladder is severely distended (pictures a, b, c).

She underwent a thorough physical examination. Her abdominal examination was unremarkable. Her digital rectal exam revealed a painless posterior rectal bulge with smooth overlying rectal mucosa. Therefore, she underwent an abdominopelvic computed tomography (CT) scan with intravenous (IV) contrast, which revealed a well-defined binoclulated cystic mass, without a soft tissue component nor calcification, measuring 11 × 10.5 × 8 cm (red arrows in Fig. 1a–d). It is located in the presacral region, in intimacy with the sigmoid colon, rectum (green arrow in Fig. 1c), ureters anteriorly (blue arrow in Fig. 1a), with both of the iliac vessels located laterally. The bladder was also severely distended (Figs. 1a–c).

Pelvic Magnetic Resonance Imaging was conducted, revealing a substantial lobulated cystic structure situated deep in the presacral and retrorectal space. The cystic formation exhibited hyperintensity on both T1 and T2 weighted images, likely attributed to mucinous or high-protein content. Fat saturation imaging (FATSTAT) did not indicate fat suppression. Notably, gadolinium-diethylenetriamine penta-acetic acid contrast injection showed no significant enhancement. The uterus, ovaries, and bladder exhibited normal findings, without evidence of pelvic lymphadenopathy or lytic/sclerotic bone lesions. Hence, a presumptive diagnosis of a tailgut cyst was made, and surgical excision of the tumor was planned.

The surgery was conducted using an anterior approach, with the patient positioned in a supine manner. The procedure commenced with a midline abdominal skin incision, followed by a layered opening. Dissection began anteriorly, where the cyst was covered by visceral peritoneum. Subsequently, the dissection proceeded superiorly and laterally on both sides of the cyst, encountering severe adhesions with adjacent structures. Meticulous dissection was carried out until the cyst was completely separated from the sigmoid, rectum, and uterus. The posterior dissection was then performed to isolate the cyst from the presacral fascia, encountering tough adhesions. Deep dissection continued until reaching the rectosacral Waldeyer’s fascia over the levator ani muscle. Thorough hemostasis and irrigation were performed during the procedure. Closure of the visceral peritoneal reflection was carried out over a pelvic drain.

**Figure 2:**
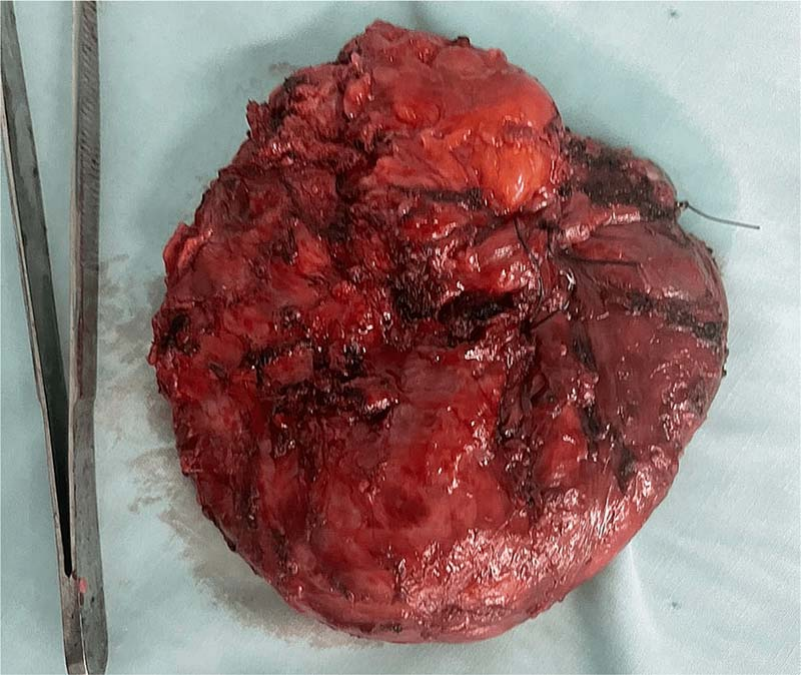
Photograph of the excised mass.

The excised mass was subjected to histopathological examination, which revealed a dark grayish cystic, thick-walled structure (Fig. 2). The cyst wall consisted of fibrovascular connective tissue containing incorporated smooth muscle and lobules resembling mature salivary acini. The surface of the cyst was lined by squamous and respiratory-type epithelium. Hence, the diagnosis of a mature cystic teratoma was established.

## Discussion

Teratomas are tumors characterized by differentiated somatic cell populations originating from germ cell layers—ectoderm, endoderm, and mesoderm—with ectodermal elements being the most common. Their benign or malignant nature depends on tissue maturity, ranging from teeth and bones to brain or gastrointestinal tissues [5]. MCTs, the most common benign germ cell neoplasm in ovaries, occur at a rate of 14.2 cases per 100 000 annually. Typically found in the uterine adnexa and the pouch of Douglas, these slow-growing tumors rarely appear in presacral locations [6, 7]. They mainly affect reproductive-aged women and have a 4.7% recurrence rate, prompting fertility-preserving surgical options [5].

Pediatric cases of tailgut cysts are rare, with limited literature available. Clinical presentations often include abdominal pain, distention, rectal pain, bleeding, dysuria, or changes in bowel habits [8–13]. A notable case involves a 12-year-old male enduring abdominal cramping for 10 years, an exceptionally prolonged instance in pediatric patients [8]. Similarly, a 12-year-old female experienced six months of lumbar pain attributable to a tailgut cyst [9], contrasting with acute presentations like pelvic pain in a 15-year-old female [10] and brief dysuria in a 14-year-old male [12]. A unique case combined a tailgut cyst with tethered cord syndrome in a 14-year-old female [14].

Histopathologically, tailgut cysts exhibit multinucleated cells, smooth muscle intertwining with fibrous tissue, inflammatory cells, salivary acini-like lobules, and ciliated epithelia [8–10, 12, 13]. Islets of Langerhans and cholesterol clefts may also be present [8, 9]. Rarely, tailgut cysts may associate with neuroendocrine tumors, as seen in a 14-year-old male patient [11]. Surgical interventions often achieve favorable outcomes, though complications like cerebrospinal fluid fistulae necessitate careful management [9]. Diagnosis challenges underscore the importance of recognizing tailgut cysts to avoid misdiagnoses such as Crohn’s disease [13].

To our knowledge, this is the first documented instance of a mature cystic teratoma mimicking a tailgut cyst in an adolescent female. We aim to raise awareness about this occurrence, reduce diagnostic delays, and improve patient outcomes.

## Data Availability

All patient-related data (history, findings, images, management, etc.) are all included in this manuscript.

## References

[1] Ebinesh A , Prakash A, Ashta A, et al. Pediatric presacral tumors with intraspinal extension: a rare entity with diagnostic challenges. Acta Radiol 2023;64:3056–73. 10.1177/02841851231202688. 37753549

[2] Hufkens A-S , Cools P, Leyman P. Tailgut cyst: report of three cases and review of the literature. Acta Chir Belg 2019;119:110–7. 10.1080/00015458.2017.1353758. 30776969

[3] Kildušis E , Samalavičius NE. Surgical management of a retro-rectal cystic hamartoma (tailgut cyst) using a trans-rectal approach: a case report and review of the literature. J Med Case Reports 2014;8:11. 10.1186/1752-1947-8-11. PMC3896874 24393234

[4] Sung M-T , Ko S-F, Niu C-K, et al. Perirenal tailgut cyst (cystic hamartoma). J Pediatr Surg 2003;38:1404–6. 10.1016/S0022-3468(03)00408-1. 14523832

[5] Cao Y , Wang B, Jia A-R, et al. Mature cystic teratoma of the ovary with a grossly visible, completely developed intestinal loop: a case report and review of the literature. Medicine (Baltimore) 2023;102:e34081. 10.1097/MD.0000000000034081. 37390246 PMC10313275

[6] Yang Y , Wang X, Li Z, et al. Identification of a mature cystic teratoma mimicking a presacral tumor by transsacral surgery in a young female: a case report. Oncol Lett 2013;6:785–8. 10.3892/ol.2013.1453. 24137411 PMC3789080

[7] Wang L , Hirano Y, Ishii T, et al. Laparoscopic surgical management of a mature presacral teratoma: a case report. Surg Case Reports 2019;5:144. 10.1186/s40792-019-0702-x. PMC6751240 31535236

[8] Cong L , Wang S, Yeung SY, et al. Mature cystic teratoma: an integrated review. Int J Mol Sci 2023;24:6141. 10.3390/ijms24076141. PMC10093990 37047114

[9] Galluzzo ML , Bailez M, Reusmann A, et al. Tailgut cyst (Retrorectal hamartoma): report of a pediatric case. Pediatr Dev Pathol Off J Soc Pediatr Pathol Paediatr Pathol Soc 2007;10:325–7. 10.2350/06-09-0166.1. 17638429

[10] Aydin Y , Tokgöz VY, Basgun N, et al. Laparoscopic management of a low-lying tailgut cyst: a rare case. J Obstet Gynaecol (Lahore) 2019;39:1181–3. 10.1080/01443615.2019.1587601. 31064225

[11] Soyer T , Aydin B, Orhan D, et al. Neuroencorine tumor arising within a tailgut cyst in an adolescent boy. Fetal Pediatr Pathol 2018;37:270–5. 10.1080/15513815.2018.1472355. 29843558

[12] Jang S-H , Jang K-S, Song Y-S, et al. Unusual prerectal location of a tailgut cyst: a case report. World J Gastroenterol 2006;12:5081–3. 10.3748/wjg.v12.i31.5081. 16937513 PMC4087420

[13] Johnson KN , Young-Fadok TM, Carpentieri D, et al. Case report: misdiagnosis of tailgut cyst presenting as recurrent perianal fistula with pelvic abscess. J Pediatr Surg 2013;48:e33–6. 10.1016/j.jpedsurg.2012.12.022. 23414899

[14] Kemp J , Guzman MA, Fitzpatrick CM, et al. Holocord syringomyelia secondary to tethered spinal cord associated with anterior sacral meningocele and tailgut cyst: case report and review of literature. Child’s Nerv Syst 2014;30:1141–6. 10.1007/s00381-014-2379-6. 24562417

